# Bioactivity of Hydrolysates Obtained from Chicken Egg Ovalbumin Using Artichoke (*Cynara scolymus* L.) Proteases

**DOI:** 10.3390/foods10020246

**Published:** 2021-01-26

**Authors:** Estefanía Bueno-Gavilá, Adela Abellán, Francisco Girón-Rodríguez, José María Cayuela, Luis Tejada

**Affiliations:** Department of Human Nutrition and Food Technology, Universidad Católica de Murcia UCAM, Campus de los Jerónimos, 30107 Guadalupe, Spain; aabellan@ucam.edu (A.A.); fgiron@ucam.edu (F.G.-R.); jmcayuela@ucam.edu (J.M.C.); ltejada@ucam.edu (L.T.)

**Keywords:** angiotensin-I converting enzyme (ACE) inhibitor, antioxidant, artichoke, bioactive peptide, ovalbumin

## Abstract

The aim of this work was to obtain chicken egg ovalbumin hydrolysates using aspartic proteinases present in extracts from the artichoke flower (*Cynara scolymus* L.) and evaluate their antioxidant, antimicrobial, and angiotensin I-converting enzyme (ACE) inhibitory activity in vitro. Hydrolysis time and molecular weight (<3 kDa) had a significant influence on the hypertensive and antioxidant activity of the hydrolysates. The <3 kDa fraction of the 16 h hydrolysate had an ACE inhibitory activity with an IC_50_ of 64.06 µg peptides/mL. The fraction <3 kDa of ovalbumin hydrolysate at 2 h of hydrolysis showed a DPPH radical scavenging activity of 30.27 µM of Trolox equivalents/mg peptides. The fraction <3 kDa of the hydrolysate of 16 h had an ABTS^+^ caption activity of 4.30 mM of Trolox equivalents/mg peptides. The fraction <3 kDa of the hydrolysate of 2 h had an iron (II) chelating activity of 32.18 µg peptides/mL. From the peptide sequences identified in the hydrolysates, we detected four peptides (from the BIOPEP database) that were already in their bioactive form (IAAEVYEHTEGSTTSY, HLFGPPGKKDPV, PIAAEVYEHTEGSTTSY, and YAEERYPIL), and are reported to display antioxidant and ACE inhibitory activity.

## 1. Introduction

Besides their elevated nutritional value, hen egg proteins or their derivatives show several biological activities, such as antioxidant activity, antihypertensive activity, antimicrobial activity, etc. [1]. Some of these proteins have activity by themselves, but the peptides derived from them, of between 2 and 20 amino acids, have shown a greater potential for bioactivity [2]. In addition to the length of the peptide chain, the amino acid composition also plays a very important role, with a higher bioactivity in peptides with hydrophobic amino acids having been observed [3]. Generally, in order for peptides to exert their bioactivity, they have to be cleaved from the sequence of their parent protein. The main procedure to obtain peptides with potential bioactivity from food proteins is enzymatic hydrolysis using different types of enzymes [4]. Generally, commercial enzymes are the most widely used because their characterization and activity are well-known [5]. Some of the enzymes most used in protein hydrolysis are pepsin, trypsin, alcalase, and papain [6,7,8,9], but the number of plant enzymes used is still limited, with enzymes of animal or microbial origin having mainly been used [10].

From the genus *Cynara* (e.g., thistle and artichoke), aspartic proteinases (cardosines and scolymines, respectively) that present high proteolytic activity can be obtained [11,12,13], giving rise to a large number of hydrophobic peptides [13,14,15] that have shown greater antioxidant [3,16,17] ACE inhibition [18] and antimicrobial activities [19].

The artichoke flower (*Cynara scolymus*) constitutes a very abundant agricultural by-product in the Spanish southeast that can be used to obtain enzymatic extracts for the production of hydrolysates with potential bioactive peptides. These hydrolysates could be used as functional food ingredients or as natural antioxidants in food systems [1,10].

Therefore, the objective of this work was to obtain ovalbumin hydrolysates with enzymatic extracts of artichoke flower. These hydrolysates have been analyzed for antioxidants, ACE-I inhibition, and antimicrobial activities in vitro. Additionally, the sequences of the peptides obtained have been identified and compared with those collected in the BIOPEP database [20].

## 2. Materials and Methods

### 2.1. Obtaining the Enzymatic Extract of Cynara scolymus L.

Mature artichoke flowers (*Cynara scolymus* L.) from the Murcia Region of Spain were used. To obtain the enzymatic extract, the methodology described by Tejada and Fernández Salguero (2003) [21] was followed. Briefly, the styles and stigmas of the artichoke flowers were trimmed and allowed to macerate in water (1:5 *w*/*v*) for 24 h. Then, the mash was sieved, and the aqueous extract was centrifuged (4000× *g* for 5 min) and filtered. The permeate obtained was lyophilized and frozen at −20 °C until use.

### 2.2. Obtaining the Hydrolysates

The hydrolysis of commercial ovalbumin (Acros Organics) with the artichoke extract was developed using the optimal conditions previously studied [15]. For this, an ovalbumin solution of 1% (*w*/*v*) in distilled water at a pH of 6.2 was prepared. The reaction was performed in tubes submerged in a shaking bath at 36 °C with an enzyme:substrate ratio of 0.0232.

Ovalbumin hydrolysates were obtained at different hydrolysis times, 2 (OH2), 4 (OH4), and 16 (OH16) h, to evaluate the effect of the hydrolysis time in obtaining peptides with bioactivity. The reaction was stopped at the corresponding time in each case, adjusting the pH to 4.5 with HCl, the isoelectric point of ovalbumin that causes its precipitation, then, it was centrifuged for 20 min at 4000× *g* and filtered with a 0.45 µm nylon filter. Finally, the hydrolysate obtained was raised to a pH of 7 with NaOH, dispensed in Falcon tubes and reserved at −20 °C until its utilization. Each type of hydrolysate was obtained in triplicate.

Furthermore, molecular weight fractions < 3 kDa were obtained from each of the hydrolysates. For this, ultrafiltration was performed by centrifugation with Amicon Ultra-15 centrifuge filters of regenerated cellulose (3000 NML; Merck Millipore, Burlington, MA, USA) at 4 °C and at 4000× *g* for 40 min. The permeate was collected in Falcon tubes at −20 °C until use.

### 2.3. Peptide Concentration

The hydrolysate peptide concentration average was obtained by the Kjeldahl nitrogen quantification method [22], using the protein conversion factor of 6.7. Prior to the digestion of the sample, the precipitation of the protein residues that could remain in the sample was carried out by adding 5% trichloroacetic acid in a 1:2 (*v*/*v*) proportion and centrifuging at 3200× *g* for 20 min. The peptide concentration was determined in triplicate for each type of hydrolysate.

### 2.4. Determination of the Hydrophobicity of Peptides

The concentration of hydrophilic and hydrophobic peptides of the hydrolysates was determined by reserve phase HPLC using the method described by González de Llano et al. (1994) [23]. The different nature of the peptides was estimated based on the retention time, considering hydrophobic peptides that appeared after the peak corresponding to the amino acid tryptophan (L-Trp ≥ 98%, Sigma-Aldrich, Madrid, Spain), and hydrophilic peptides that appeared in the portion of peptides retained between the amino acid tyrosine (L-Tyr, +99%, Acros Organics, Fairlawn, NJ, USA) and the amino acid tryptophan. These determinations were conducted in triplicate for each type of hydrolysate.

### 2.5. Angiotensin I-Converting Enzyme Inhibitory Activity

The angiotensin I-converting enzyme (ACE) inhibitory activity was determined by the method based on the spectrophotometric technique of Cushman and Cheung (1971) [24] adapted by Miguel et al. (2004) [25].

One hundred µL of substrate solution consisting of 5 mM of hipuryl-histidyl-leucine (HHL; Sigma-Aldrich), dissolved in 0.1 M of borate buffer and 0.3 M of NaCl at a pH of 8.3, was blended with 40 µL of hydrolysate at a concentration of 125 µg of peptides/mL. Two mU of ACE (EC 3.4.15.1., Sigma-Aldrich) was added, and the reaction was incubated for 30 min at 37 °C. After incubation, the enzyme was inactivated with 150 µL of HCl 1N. The hippuric acid formed in the reaction was extracted by adding 1000 µL of ethyl acetate. The blend was centrifuged at 4000× *g* for 10 min, and 800 µL of the organic phase was taken, which was subsequently placed on a heating plate for evaporation. The residue of hippuric acid formed was resuspended in 1000 µL of distilled water, and the absorbance at 228 nm was measured. The ACE inhibition percentage was determined with the following formula:

ACE inhibitory activity (%) = ((Acontrol − Ablank) − (Asample − Ablank))/(Acontrol − Ablank)) × 100(1)

where Acontrol is the absorbance of hippuric acid formed during the reaction without inhibiting substances, Asample is the absorbance of hippuric acid formed in the reaction in the presence of the hydrolysate, and Ablank is the absorbance of HHL that has not reacted in distilled water. The assay was carried out in triplicate for each type of hydrolysate.

The half-maximal inhibitory concentration (IC_50_) value is defined as the concentration of peptides required to inhibit 50% of the ACE activity. It was calculated for OH16, as well as its <3 kDa fraction using the concentration range of 25–125 µg peptides/mL. The IC_50_ value was obtained in triplicate.

### 2.6. Antioxidant Activity

#### 2.6.1. DPPH Radical Scavenging Activity

The DPPH (2,2-diphenyl-1-picrylhydrazyl (Sigma-Aldrich)) radical scavenging activity (RSA) was analyzed following the method described by Bersuder et al. (1998) [26], with slight modifications; 500 µL of the hydrolysate was added to 625 µL of DPPH 0.004% (*w/v*) in ethanol. The mixture was allowed to react for one hour in the dark at room temperature, and then it was centrifuged at 10,000× *g* for 2 min. The absorbance of the supernatant was measured at 517 nm. The percentage of antioxidant activity was determined applying this following formula:

DPPH RSA (%) = [((Acontrol − Ablank) − (Asample − Ablank))/(Acontrol − Ablank)] × 100(2)

where Acontrol is the absorbance of the DPPH with water instead of hydrolysate, Asample is the absorbance of the radical DPPH in the presence of the hydrolysate, and Ablank is the absorbance of the blank (containing 50% of ethanol in distilled water (*v/v*)). The antioxidant activity against DPPH was calculated as the Trolox equivalent antioxidant capacity (TEAC; µM Trolox/mg of peptides). The experiment was conducted in triplicate for each type of hydrolysate.

#### 2.6.2. ABTS Radical Scavenging Activity

The ABTS ((2,2′-azino-bis-(3-ethylbenzothiazoline-6-sulfonic acid) (Sigma, Aldrich)) radical scavenging activity of the hydrolysates was determined according to the method conducted by De Gobba et al. (2014) [27]. To create the radical (ABTS^+^), a solution of ABTS (19.4 mM) and potassium persulfate (6.7 mM) in distilled water was allowed to react overnight. The ABTS^+^ solution was reduced approximately 350 times with 10 mM of phosphate buffer at a pH of 7.4 to reach an absorbance measure range at 405 nm of 0.6 to 0.7.

To 200 µL of the ABTS^+^ working solution, 50 µL of hydrolysate (at different concentrations) was added, and the absorbance was measured for 30 min. The last measurements were used to calculate the percentage of antioxidant activity applying the following formula:

ABTS RSA (%) = 100 − [100 × ((Asample − Ablank)/(Acontrol − Ablank))](3)

where Acontrol is the absorbance of the ABTS^+^ solution with water instead of the sample, Abs sample is the absorbance of the ABTS^+^ solution with hydrolysate, and Ablank is the absorbance of the blank (containing only the phosphate buffer). The half-maximal scavenging concentration (SC_50_) was the peptide concentration necessary to scavenge 50% of the ABTS^+^. The SC_50_ was obtained using the sample concentration range of 2–20 µg of peptides/mL. The TEAC value of the hydrolysates (mM of Trolox equivalents/mg of peptides) was also assessed. The assay was conducted in triplicate for each type of hydrolysate.

#### 2.6.3. Iron (II) Chelating Activity

The iron (II) chelating activity was determined according to the method described by Wu et al. (2007) [28], with some modifications; 100 µL of 75 µM Cl_2_Fe solution in distilled water was mixed with 25 µL of the hydrolysate. The blend was incubated at room temperature for 10 min. Then, 100 µL of 500 µM Ferrozine (3-(2-pyridyl)-5,6-dipjenyl-1,2,4-triazine-p,p′-disulfonic acid) (Sigma, Aldrich) solution in water was added, and the absorbance was measured at 560 nm. The inhibition of the iron–Ferrozine complex formation was calculated according to the following formula:

Iron chelating activity (%) = 100 − (100 × (Asample − Ablank)/(Acontrol − Ablank))(4)

where Acontrol is the absorbance of the ferrozine complexed with iron without the hydrolysate, Asample is the absorbance of the ferrozine complexed in the presence of the hydrolysate, and Ablank is the absorbance of the blank (containing only distilled water). IC_50_ is defined as the peptide concentration necessary to inhibit 50% of the iron–Ferrozine complex formation. IC_50_ was calculated using a range of peptide concentrations of 11–83 µg peptides/mL. The assay was conducted in triplicate for each type of hydrolysate.

### 2.7. Antimicrobial Activity

Antimicrobial activity was determined by comparing the bacterial growth curves following the method of Hill et al. (2013) [29], with some modifications described by Bueno-Gavilá et al. (2019) [30]. The bacterial species studied were *Enterococcus faecalis* (NCIMB 775), *Escherichia coli* (NCIMB 9484), *Listeria innocua* (CCUG 15531), and *Pseudomonas fluorescens* (NCIMB 9046). All of these species were acquired from the Spanish Type Culture Collection. The culture media used were peptone water (Pareac-Cultimed, Barcelona, Spain), tryptic soy broth (Scharlau, Sentmenat, Spain), and plate count agar (Scharlau). As a positive inhibition control, gentamicin (Sigma-Aldrich) of ≥95% purity was used. The curve data were processed using DMFit 3.5 according to the model of Baranyi and Roberts (1994), obtaining the most representative kinetic parameters (latency phase and maximum growth rate) and the maximum growth. The experiment was carried out in triplicate for each type of hydrolysate.

### 2.8. Peptide Identification

The identification of the peptides in the hydrolysates and the analysis of the bioactivity of these peptides identified were performed in the Proteomics and Bioinformatics units of the University of Córdoba (Spain). The peptide identification was carried out through nano-liquid chromatography-tandem MS analysis, performing a previous digestion with trypsin, following the methodology described in Bueno-Gavilá et al. 2019 [30].

For the analysis of the bioactivity in the peptides identified, the database of bioactive peptides, BIOPEP [20], was used. Two types of searches were carried out: the identification of the peptides in their bioactive form in the sample, and the identification of peptides with potential bioactivity, because they contain bioactive sequences in their primary structure. The analysis of the data obtained was executed using R (version 3.4.0; https://www.r-project.org).

### 2.9. Statistical Analysis

Statistical analyses were performed using the SPSS program (version 21; IBM Corporation, Armonk, NY, USA). We performed ANOVA and Tukey tests to determine the hydrolysis time and molecular weight effect in the various defined parameters. In the study of the antibacterial activity of the hydrolysates, we used one-way ANOVA and the Dunnet test to identify differences between the means of the samples and the control, setting a confidence level of 95%.

## 3. Results

### 3.1. Total Peptides and Hydrophilic and Hydrophobic Peptides of Ovalbumin Hydrolysates

The concentrations of each of the total hydrolysates (THs) and their fractions of molecular weight < 3 kDa are shown in Table 1. The hydrolysis time significantly affected the peptide concentration of the hydrolysates (*p* < 0.01), OH16 being the one that presented the highest concentration of peptides both in the TH and in its fraction < 3 kDa.

The hydrolysis time significantly affected the ratio of hydrophobic (HO) and hydrophilic (HI) peptides (HO/HI) of the ovalbumin hydrolysates (*p* < 0.05). The OH2, both in its TH and in the <3 kDa fraction, was the one with the highest HO/HI ratio (4.01 ± 0.25% and 2.68 ± 0.06%, respectively). There were no significant differences in the HO/HI ratio between the OH4 and OH16 for both the THs and their <3 kDa fractions.

### 3.2. ACE Inhibitory Activity

**Figure 1:**
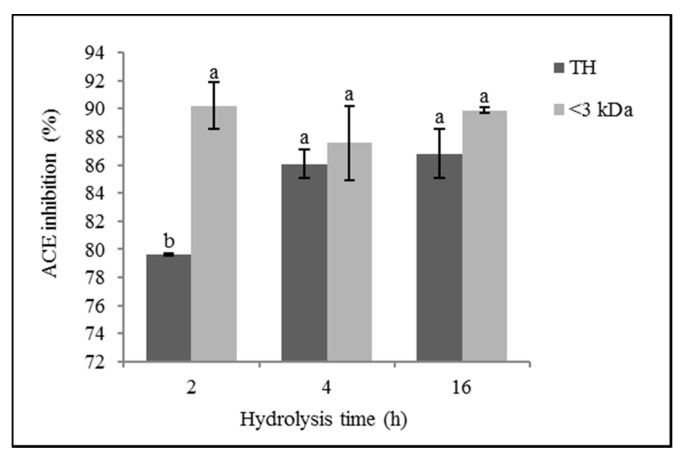
Angiotensin I-converting enzyme (ACE) inhibitory activity (%) of ovalbumin total hydrolysates (THs) and their low molecular weight fractions (<3 kDa). Values are means ± SEs (*n* = 3). Bars with distinct letters (a,b) were significantly different (LSD test, *p* < 0.05).

Figure 1 shows the ACE inhibitory activity percentages of the ovalbumin hydrolysates with *Cynara scolymus* extract, both of the THs and their <3 kDa fractions at a concentration of 125 µg/mL.

The hydrolysis time in general did not significantly influence the ACE inhibitory activity of ovalbumin hydrolysates (*p* > 0.05), although TH, OH4, and OH16 showed greater activity (86.07 ± 0.99% and 86.78 ± 1.75%, respectively) than OH2 (79.64 ± 0.06%) (*p* ≤ 0.05). In the molecular weight fractions < 3 kDa, there were no significant differences in the ACE inhibitory activity between the different hydrolysis times (*p* > 0.05).

The molecular size of the peptides had a significant influence on the ACE inhibitory activity (*p* ≤ 0.01), with the <3 kDa fractions of the hydrolysates showing the highest percentage of inhibition in all of the cases, although no significant statistical differences (*p* > 0.05) were observed between the ACE inhibitory activity of the OH4 TH and the OH16 TH (86.07 ± 0.99% and 86.78 ± 1.75%, respectively) and their fractions <3 kDa (87.55 ± 2.63% and 89.87 ± 0.21%, respectively).

OH16 and its fraction <3 kDa had an IC_50_ value of 69.55 ± 3.12 µg/mL and 64.04 ± 0.37 µg/mL, respectively.

### 3.3. Antioxidant activity

#### 3.3.1. DPPH Radical Scavenging Activity

**Figure 2:**
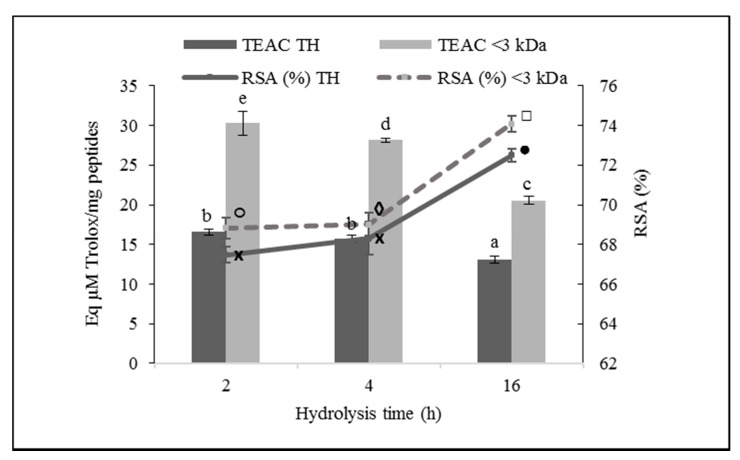
2,2-Diphenyl-1-pycrylhydrazyl (DPPH) radical scavenging activity (RSA) of total ovalbumin hydrolysates (THs) and their low molecular weight fractions (<3 kDa). Bars show the Trolox equivalent antioxidant capacity (TEAC; µM of Trolox equivalents per mg of peptides), and lines represent the percentage of RSA. Values are means ± SEs (*n* = 3). Bars with distinct letters (a–e) and lines with distinct symbols (●, ×, □, ◊, ○) were significantly different (LSD test, *p* < 0.05).

The results of the antioxidant activity of ovalbumin hydrolysates against the DPPH radical are shown in Figure 2. The hydrolysis time significantly affected the antioxidant activity of the hydrolysates against the DPPH radical (*p* ≤ 0.01).

Taking into account the percentage of DPPH RSA of the hydrolysates, the activity increased with the hydrolysis time (*p* ≤ 0.05). This must be due to the higher peptide concentration of the hydrolysates at a longer hydrolysis time. Paying attention to the Trolox (µM) equivalent antioxidant capacity per mg of peptides, the antioxidant capacity decreased with the hydrolysis time, with OH2 being the most powerful hydrolysate in the uptake of the DPPH radical, with a TEAC value of 16.54 µM of Trolox/mg of peptides.

The molecular weight of the peptides also significantly affected the DPPH RSA of the ovalbumin hydrolysates (*p* ≤ 0.05), with the fraction <3 kDa being more potent than the TH at the three hydrolysis times. Considering the percentage of DPPH RSA, OH16 < 3 kDa was the sample that presented the highest activity, with 74.09 ± 0.40% of radical caption, however, attending at TEAC, OH2 < 3 kDa was the most powerful sample, with a value of 30.27 ± 1.51 µM of Trolox/mg of peptides.

#### 3.3.2. ABTS Radical Scavenging Activity

**Figure 3:**
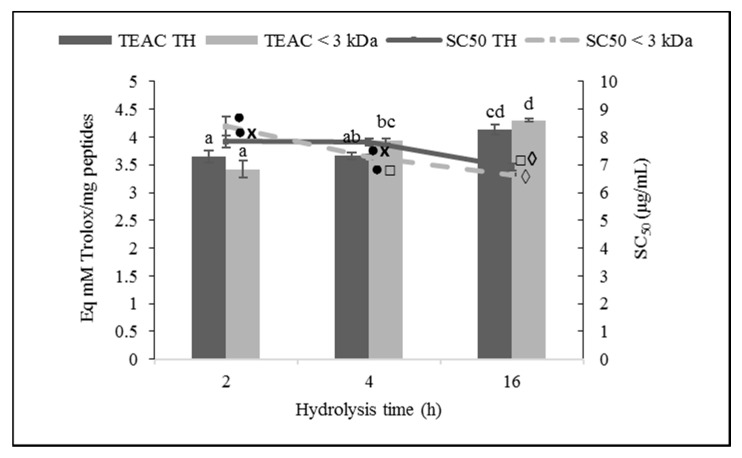
2,2′-Azino-bis-(3-ethylbenzothiazoline-6-sulfonic acid) (ABTS) radical scavenging activity (RSA) of ovalbumin total hydrolysates (THs) and their low molecular weight fractions (<3 kDa). Bars show the Trolox equivalent antioxidant capacity (TEAC; mM of Trolox equivalents per mg of peptides), and lines represent the half-maximal scavenging concentration (SC_50_; µg/mL). Values are means ± SEs (*n* = 3). Bars with distinct letters (a–d) and lines with distinct symbols (●, ×, □, ◊) were significantly different (LSD test, *p* < 0.05).

Hydrolysis time significantly affected the antioxidant activity of ovalbumin hydrolysates with *C. scolymus* L. against the ABTS radical (*p* ≤ 0.01), producing a decline in the IC_50_ value and, concurrently, a rise in the TEAC, indicating a higher antioxidant power of the hydrolysates (Figure 3). Thus, OH16 and its fraction <3 kDa was the hydrolysate with the highest antioxidant capacity against the ABTS radical (*p* ≤ 0.05), presenting an IC_50_ of 6.92 ± 0.14 and 6.65 ± 0.04 µg/mL, respectively. Nonetheless, molecular size did not affect the ABTS RSA of the ovalbumin hydrolysates (*p* > 0.05).

#### 3.3.3. Iron (II) Chelating Activity

**Figure 4:**
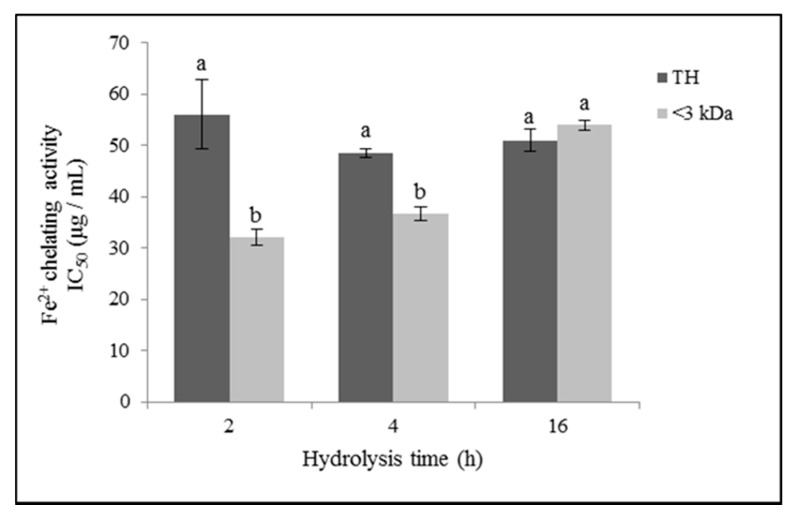
Iron (II) chelating activity (half-maximal inhibitory concentration (IC_50_); µg/mL)) of ovalbumin total hydrolysates (THs) and their low molecular weight fractions (<3 kDa). Values represent means ± SEs (*n* = 3). Bars with distinct letters (a–b) were significantly different (LDS test, *p* < 0.05).

The iron (II) chelating capacity of the ovalbumin hydrolysates obtained with the artichoke flower extract is shown in Figure 4. Regarding the effect of the hydrolysis time in the iron chelating activity of the TH, no significant differences were observed (*p* ≥ 0.05). Nonetheless, considering the < 3 kDa fractions of the hydrolysates, the chelating capacity decreased significantly with the hydrolysis time (*p* ≤ 0.05), with OH2 < 3 kDa being the sample that presented the highest Fe^2+^ chelating activity, with an IC_50_ value of 32.18 µg/mL. Precisely, OH2 < 3 kDa presented the highest ratio of HO/HI peptides. The molecular weight of the peptides significantly affected the iron (II) chelating activity (*p* ≤ 0.01), with the <3 kDa fractions showing the highest chelating potency (*p* ≤ 0.05). However, in the case of OH16, no significant differences (*p* > 0.05) were found between TH and its fraction <3 kDa.

### 3.4. Antimicrobial Activity

Analyzing the effect of the hydrolysates on the growth of the Gram negative microorganisms tested, only a statistically significant inhibitory effect was observed (*p* ≤ 0.05) for the growth rate of *E. faecalis* with OH2 TH and with the <3 kDa fractions at the different hydrolysis times (Table 2). However, the lag phase was shortened (*p* ≤ 0.01), and there were no changes in the maximum growth of the microorganism (*p* > 0.05). Regarding *E. coli*, no antimicrobial effect of the hydrolysates was detected on the growth curves. There was no antimicrobial effect of the hydrolysates against the Gram positive microorganisms tested, *L. innocua* and *p. fluorescens* (data not shown).

### 3.5. Identification of the Bioactive Peptides Present in the Oovalbumin Hydrolysates

Table 3 shows the sequences of peptides from the ovalbumin hydrolysates, in their bioactive form, that have demonstrated biological activity. The identified peptide sequences common to the three types of ovalbumin hydrolysates (2, 4, and 16 h) that have demonstrated bioactivity are IAAEVYEHTEGSTTSY with antioxidant activity [31] and YAEERYPIL with ACE inhibitory activity [32]. On the other hand, non-shared peptides have been identified in OH2 and H4: PIAAEVYEHTEGSTTSY with antioxidant activity [31] and HLFGPPGKKDPV with ACE inhibitory activity [33], respectively.

**Figure 5:**
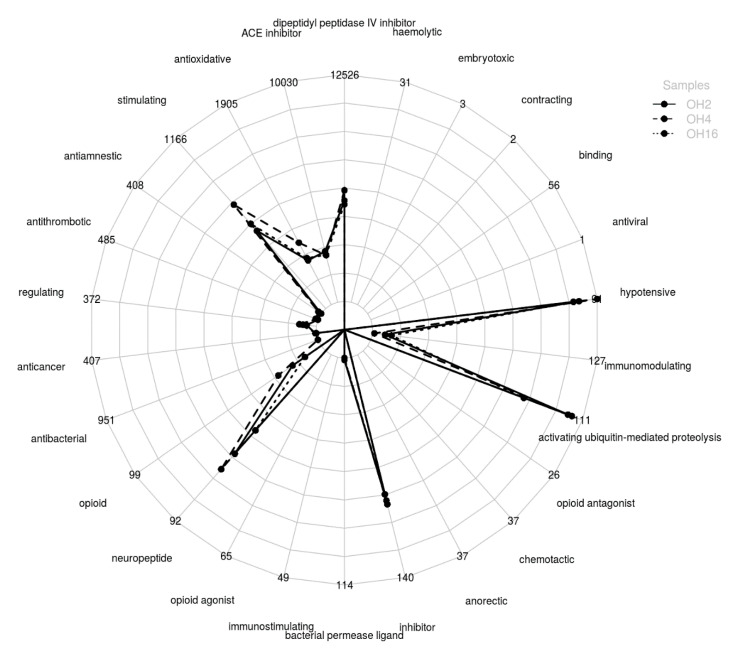
Quantification of potential bioactive peptides in ovalbumin total hydrolysates using *Cynara scolymus* water-soluble extract at different hydrolysis times: 2 h (OH2), 4 h (OH4), and 16 h (OH16), considering their peptide spectral matches (PSMs) in liquid chromatography mass spectrometry and taking into account the presence of demonstrated bioactive sequences in their primary structure. The identified peptides and their respective quantification for each sample were extracted. The quantification values were normalized according to the total PSM of all peptides in the samples. In this way, quantification of the same peptide between samples was comparable. ACE = angiotensin I-converting enzyme.

On the other hand, in the ovalbumin hydrolysates with *C. scolymus* L. flower extract, peptides with possible potential biological activity have been identified, since encrypted sequences that have demonstrated bioactivity have been found within their primary structure. Figure 5 shows the distribution of the peptides with putative activity present in the ovalbumin hydrolysates at the three hydrolysis times. At all hydrolysis times, peptides containing sequences with bioactivity were obtained. OH4 yielded the highest amount of peptides with fragments with bioactivity, highlighting antioxidant activity, hypotensive activity, inhibition of dipeptidyl peptidase IV activity, activation of proteolysis mediated by ubiquitin activity, stimulant activity, opioid activity, and peptides with sequences that work as neuropeptides.

## 4. Discussion

### 4.1. Total Peptides and Hydrophilic and Hydrophobic Peptides of Ovalbumin Hydrolysates

Our results are consistent with several studies on egg white protein hydrolysates, where the degree of hydrolysis increased with hydrolysis time, using both enzymes of plant origin, such as papain [34,35], as well as digestive enzymes of animal origin [25,34,36], microbial origin (such as alcalase, neutrase, and thermolysin) [34,37,38], or fungal origin (Flavourzyme) [34].

No studies have been found regarding the total concentration and proportion of hydrophobic and hydrophilic peptides in egg white hydrolysates. However, some publications have reported the amino acid composition of the hydrolysates and, occasionally, their percentages of hydrophobic amino acids [16,39,40,41]. Normally, the content of hydrophobic amino acids and their hydrophobic value determines the hydrophobicity of the hydrolysates [16]. The OH2 presented a hydrophobic peptide concentration of 80.02 ± 5.64% in the TH and of 72.83 ± 1.91% in its fraction <3 kDa. These values are higher than those indicated in different studies on egg white protein hydrolysates. Thus, Ren et al. (2014a) obtained 48.51% of hydrophobic amino acids in a duck egg white hydrolysate, elaborated with an enzymatic combination, with a specific hydrolase for egg protein and alcalase [16]. Sun et al. (2014) reported 43.23% of hydrophobic amino acids in the 2–5 kDa fraction of an egg white hydrolysate made with pepsin [40]. Liu et al. (2015) determined 42.76% of hydrophobic amino acids in the <1 kDa fraction of an egg white hydrolysate with alcalase [42]. Chen et al. (2012) obtained percentages of hydrophobic amino acids of 44.93 and 49.28% for the TH of egg white with pepsin and its fraction < 3 kDa, respectively [39]. Chen and Chi (2011) indicated 46.97% of hydrophobic amino acids in their egg white hydrolysate prepared with papain at the hydrolysis time of three hours [35]. In the same way, You and Wu (2011) elaborated different hydrolysates from egg white protein with thermolysin, alcalase, and a combination of pepsin and pancreatin at three hours of hydrolysis time, obtaining 44.46%, 43.55%, and 44.80% of hydrophobic amino acids for each of them, respectively [41]. Due to the high proportion of hydrophobic peptides in our hydrolysates, it is expected that they yield high ACE inhibitory, antioxidant, and antimicrobial activity [19,25,43,44].

### 4.2. ACE Inhibitory Activity

The results observed are related to those reported by various authors who observed that there is an optimal degree of hydrolysis in the formation of a high concentration of peptides with ACE inhibitory activity [39,45]. Thus, Chiang et al. (2008) described that after two to four hours of hydrolysis time, the inhibitory activity of ACE was stabilized [38]. Tanzadehpanah et al. (2013) described that, in the first hydrolysis intervals, the ACE inhibitory activity of the hydrolysates rose until achieving its maximum at four hours of hydrolysis [46]. Chen et al. (2012b) obtained a maximum ACE inhibitory activity at five hours of hydrolysis [39], and Quirós et al. (2007) described that the ACE activity of the hydrolysates increased with the hydrolysis time up to eight hours [45].

The <3 kDa fractions of the hydrolysates showed the greatest inhibition of the ACE. These results are consistent with those observed in other studies, where lower molecular weight peptides were attributed the main ACE inhibitory activity of hydrolysates [25,38].

OH16 and its fraction <3 kDa had an IC_50_ value of 69.55 ± 3.12 µg/mL and 64.04 ± 0.37 µg/mL, respectively. These values are lower (indicating greater activity) than those reported for other ovalbumin hydrolysates using proteases of plant origin. For example, in the hydrolysis of egg white with the commercial protease Promod 144P from *Carica papaya*, an optimal IC_50_ of 78.7 µg/mL was obtained at 24 h of hydrolysis [34]. Pokora et al. (2014) prepared egg white protein hydrolysates with proteases extracted from *Cucurbita ficifolia*, obtaining an ACE inhibitory activity after five hours of hydrolysis, with an IC_50_ of 9071 µg/mL [47]. Similarly, Chen and Chi (2011) obtained an egg white protein hydrolysate elaborated with papain at a hydrolysis time of three hours that yielded an IC_50_ of 1676 µg/mL [35], this concentration being 24 times higher than that observed in OH16. 

In the same way, several works that used animal or microbial origin proteases obtained less ACE inhibitory activity than that shown by our hydrolysates prepared with artichoke extract. For example, Huang et al. (2015) reported a 70.55% ACE inhibition in ovalbumin hydrolysates with pepsin at a hydrolysis time of approximately four hours [36]. Abeyrathne et al. (2014) used combinations of alcalase, papain, pepsin, trypsin, and chymotrypsin, obtaining less than 80% inhibition in all cases [48]. Tanzadehpanah et al. (2013) used trypsin for the production of ostrich egg white hydrolysates, reporting ACE inhibition percentages between 28 and 57% [46]. In ovalbumin hydrolysates with pepsin obtained at three hours of hydrolysis, an IC_50_ of 643.1 µg/mL was reported [49], this concentration being about nine times higher than that obtained for OH16. Chen et al. (2012b) observed the highest ACE inhibitory activity (50.61%) at five hours of hydrolysis of egg white with trypsin [39], using a hydrolysate concentration of 1 mg/mL, eight times higher than that used in our assay (0.125 mg/mL).

The ovalbumin hydrolysates prepared with *Cynara scolymus* L. aqueous flower extract, even at short periods of hydrolysis, presented a powerful inhibitory activity of ACE in vitro compared to those described in other studies using other enzymes of plant, animal, and microbial origin, demonstrating the importance of the specificity of the proteinase used in the production of bioactive peptides [50,51,52]. Thereby, the aspartic proteinases of flowers of various species of the genus *Cynara* proved to have an elevated proteolytic activity that results in deep fragmentation [53], producing small peptides that are more accessible to the active site of the ACE [54]. Furthermore, our hydrolysates obtained with artichoke flower extract presented a high concentration of hydrophobic peptides, which may explain their greater ACE inhibitory activity [18]. Nevertheless, the influence of the food protein used as a substrate must be taken into account. In fact, in a previous study using the *Cynara scolymus* enzyme extract over bovine casein, a lower ACE inhibition in the hydrolysates (with an IC_50_ of 114.21 µg/mL at a hydrolysis time of 16 h) than that showed by ovalbumin hydrolysates was obtained, which suggests that the hydrolysis of ovalbumin with cynarases yields peptides with greater potential antihypertensive activity than those obtained from bovine casein [30].

### 4.3. Antioxidant Activity

Antioxidant peptides from eggs could avoid oxidative harm through numerous routes, such as free radical scavenging, chelating pro-oxidative transition metal ions, inactivation of reactive oxygen species, and reducing hydroperoxides [4,55,56], and therefore, it is necessary to evaluate their antioxidant activity with different assays.

Several studies with egg white protein hydrolysates reported a dose-dependent DPPH RSA, increasing this activity with the hydrolysate concentration [35,39,57]. According to TEAC values, OH2 showed the most powerful DPPH RSA. This may be due to the fact that the greater amount of aromatic and hydrophobic amino acid residues in the peptides could grant greater antioxidant power to the hydrolysates, since these amino acids supply hydrogen to the reactive oxygen species [44]. OH2 was precisely the hydrolysate that presented the highest ratio of hydrophobic peptides. From this point of view, in antioxidant capacity tests with fat-soluble systems, such as the DPPH radical one, the hydrophobicity of the peptides is a relevant point in the free radical scavenging activity [58]. The molecular weight of the peptides also significantly affected the DPPH RSA. Similarly, Chen et al. (2012a) described better activity in the fraction <3 kDa (78.74%) than in the total (73.14%) of egg white protein hydrolysates prepared with papain during three hours of hydrolysis at 5 mg/mL [57], this concentration being about five times higher than that used in our assays. Our results also showed greater activity against DPPH than those reported for the fraction <1 kDa of an alcalase egg white protein hydrolysate with a 39.51% RSA [42]. The ovalbumin hydrolysates elaborated with *Cynara scolymus* extract showed a significantly higher DPPH RSA (*p* < 0.01) than those obtained from bovine casein, both in TH and <3 kDa fractions (30.89% and 25.67%, respectively, in casein hydrolysates vs. 72.51% and 74.09%, respectively, in ovalbumin hydrolysates) [30].

The ovalbumin hydrolysates produced with proteases extracted from the artichoke flower displayed a powerful antioxidant activity against the ABTS radical. Thus, OH16 gave an IC_50_ value (6.92 ± 0.14 µg/mL) about 51 times lower (indicating greater activity) than that reported for a hydrolyzed ostrich egg white with pepsin and pancreatin (0.36 mg/mL) [59]. In the same way, Noh and Suh (2015) described the decrease in IC_50_ with hydrolysis time in liquid egg white hydrolysates prepared with alcalase, achieving the lowest IC_50_ value at 32 h of hydrolysis time (0.84 mg/mL) [60], this concentration being approximately 120 times higher than that showed by OH16 with artichoke flower extract. On the other hand, taking into consideration the activity of the artichoke extract in other food matrices, in the case of ABTS^+^ RSA, there were no statistically significant differences between the ovalbumin hydrolysates and the bovine casein at the same hydrolysis times (*p* > 0.05) [30].

The ovalbumin hydrolysates with artichoke flower extract showed an iron (II) chelating activity more potent than that described by Baratzadeh et al. (2013) for two peptides purified from goose egg white protein hydrolysate with papain, yielding an IC_50_ > 100 µg/mL [61]. In addition, comparing the activity of the artichoke extract to bovine casein, there was a significantly lower iron (II) chelating activity in this substrate (*p* < 0.01) in both the TH and <3 kDa fraction of the hydrolysates at the same hydrolysis time (16 h), showing that the casein hydrolysates had an IC_50_ value much higher (337.09 µg/mL in the TH, and 221.49 µg/mL in its <3 kDa fraction) [30].

The ovalbumin hydrolysates produced with artichoke flower extract proteases showed great differences between the different antioxidant activity tests. The activity of the hydrolysates against the DPPH radical was substantially lower than that shown in the inhibition of the ABTS radical, with TEAC values of µM of Trolox/mg vs. mM of Trolox/mg, respectively. These results are similar to those obtained in other works with enzymatic hydrolysates of egg white protein [37,42,60]. This difference is probably due to the distinct solubility and diffusion of the radicals used [62]. DPPH, being a fat-soluble compound despite being dissolved in alcohol, may not disperse well to the target peptides in an aqueous solution, thus, its interaction could be restricted. On the contrary, radical species soluble in water, such as ABTS^+^, could be more accessible to peptides in an aqueous solution, and thus react more efficiently with the peptides of hydrolysates [60]. In addition, with the longer hydrolysis time, a higher amount of amino acids and small peptides are produced, which are hydrophilic, and therefore accomplish the scavenging reaction more easily with the ABTS radical than with DPPH [62]. Thus, we have seen that the activity of our hydrolysates against the ABTS radical increased with the hydrolysis time, exactly the opposite of what happened when it was against the DPPH radical. However, the importance of hydrophobic amino acids in free radical scavenging has been described [63], so that peptides with an elevated amount of hydrophobic amino acids could raise the antioxidant activity of hydrolysates. In this sense, the two-hour hydrolysate, with the highest ratio of OH/HI peptides, yielded better iron chelating and DPPH radical scavenging activity.

### 4.4. Antimicrobial Activity

Not many studies have been found in the literature about antimicrobial activity in hydrolysates of ovalbumin of chicken egg white or other poultry species. Most of the studies found on antimicrobial activity in egg hydrolysates are based on the hydrolysis of lysozyme from egg white. Lysozyme is an enzyme that has demonstrated antimicrobial activity by itself, being capable of hydrolyzing the β bonds between N-acetylneuramic acid and N-acetyl glucosamine of bacterial walls [64]. Memarpoor-Yazdi et al. (2012) isolated the NTDGSTDYGILQINSR peptide from the hydrolysis of hen egg white lysozyme with an enzymatic combination of papain and trypsin. This peptide showed antibacterial activity against *E. coli* and *L. mesenteroides* [65]. Furthermore, Thammasirirak et al. (2010) isolated the TAKPEGLSY peptide from the hydrolysis of goose egg white lysozyme with pepsin and trypsin, which was shown to have activity against *V. cholerae* and *S. epidermidis* [66]. Similarly, Mine et al. (2004) isolated two antimicrobial peptides from hen egg white lysozyme by digestion with pepsin and trypsin, IVSDGDMNAW and HGLDNYR, with activity against *E. coli* K-12 and *Staphylococcus aureus* 23–394 [67]. On the other hand, Tang et al. (2013) described antimicrobial activity in different ovalbumin hydrolysates obtained with pepsin, trypsin, and papain at determined hydrolysis times, since the same enzyme, depending on the time it was allowed to act on the substrate, could give rise to peptides, which, instead of inhibiting, enhanced the growth of the microorganism. In this same study, in the case of ovalbumin hydrolysates obtained with Flavourzyme^®^, neutrase, and alcalase, at any of the different hydrolysis times tested (one, two, three, four, and five hours), the growth of *E. coli* was promoted, which could indicate that the hydrolysis time, which affects the degree of cleavage of the peptide chain, or the specificity of certain enzymes, can provide the hydrolysate with nutrients that stimulate the growth of the microorganism [68]. Regarding the enzymatic artichoke extract activity producing bovine casein hydrolysates, we obtained peptides that gave similar antimicrobial results to those obtained with the ovalbumin hydrolysates, showing only a slight inhibition on the growing of *E. faecalis*, increasing its Lag phase and lowering its maximum growth, but having no influence in the other microorganisms tested [30].

### 4.5. Peptide Identification

A large number of peptides with multiple in vitro biological activities have also been identified from egg white protein. For example, WESLSRLLG peptide obtained from the hydrolysis of ostrich egg white protein with pepsin and pancreatin demonstrated free radical scavenging activity and ACE inhibition (IC_50_: 47.6 µg/mL) [69]; the peptides FRADHPFL, RADHPFL, YAEERYPIL, YRGGLEPINF, ESIINF, RDILNQ, IVF, YQIGL, SALAM, and FSL, obtained from the hydrolysis of hen egg white with pepsin, have shown ACE inhibition, as well as antioxidant, vasodilator, and antihypertensive activity [34]; DHTKE, FFGFN, and MPDAHL, from the hydrolysis of egg white protein with alkaline, demonstrated antioxidant activity [42]. YAEERYPIL sequence was identified in the egg white hydrolysate with pepsin [34] and, precisely, this ACE inhibitor peptide has also been identified in the ovalbumin hydrolysates with artichoke flower extract.

## 5. Conclusions

Ovalbumin peptides obtained from extracts of *C. scolymus* demonstrated an elevated level of ACE inhibition and antioxidant activity in vitro compared with the findings of similar studies using enzymes of different origins (vegetable, animal, or bacterial). However, the hydrolysates did not show antibacterial activity in vitro. The hydrolysis time of 16 h yielded hydrolysates with a greater concentration of peptides. The hydrolysate obtained at a hydrolysis time of two hours showed a higher concentration of hydrophobic peptides, its low molecular weight fraction of <3 kDa being the sample that demonstrated a greater level of ACE inhibition, DPPH radical scavenging activity, and iron (II) chelating activity. We identified the sequence of four bioactive peptides that demonstrated antioxidant and ACE inhibition activities. We also identified a large number of peptides with putative activity and that possessed fragments of bioactive sequences, particularly sequences related to antioxidant activity, hypotensive activity, and inhibition of dipeptidyl peptidase IV.

## Figures and Tables

**Table 1:** Effect of hydrolysis time on the peptide concentration and hydrophobicity of ovalbumin hydrolysates from *Cynara scolymus* proteases. ^1^

Item ^2^		Hydrolysis Time (H)			*p*-Value ^3^		
		2	4	16	H	MW	H × MW
Peptides	TH	2.35 ± 0.07 ^c^	2.50 ± 0.04 ^d^	3.21 ± 0.10 ^e^	≤0.01	≤0.01	>0.05
(mg/mL)	<3 kDa	1.31 ± 0.07 ^a^	1.41 ± 0.00 ^a^	2.08 ± 0.04 ^b^			
HI (%)	TH	19.98 ± 0.45 ^b^	23.27 ± 0.17 ^c^	25.12 ± 4.18 ^c^	≤0.01	≤0.01	>0.05
	<3 kDa	27.17 ± 0.54 ^a^	44.32 ± 0.97 ^b^	50.71 ± 2.43 ^b^			
HO (%)	TH	80.02 ± 5.64 ^b^	76.73 ± 6.03 ^c^	74.88 ± 9.92 ^b^	≤0.05	≤0.01	≤0.05
	<3 kDa	72.83 ± 1.91 ^a^	55.68 ± 0.97 ^a^	49.29 ± 8.75 ^a^			
HO/HI	TH	4.01 ± 0.25 ^d^	3.30 ± 0.23 ^bc^	2.98 ± 0.98 ^bc^	≤0.05	≤0.01	>0.05
	<3 kDa	2.68 ± 0.06 ^b^	1.26 ± 0.04 ^a^	0.97 ± 0.13 ^a^			

^a–d^ Values within a row with distinct superscript letters were significantly different (LSD test, *p* < 0.05). ^1^ Values represent the mean ± SE (*n* = 3). ^2^ Less than 3 kDa = hydrolysate fraction on molecular eight < 3 kDa; HI = hydrophilic peptides; HO = hydrophobic peptides; HO/HI = hydrophobic and hydrophilic peptide ratio. ^3^ H = hydrolysis time; MW = molecular weight; H × MW = hydrolysis time × molecular weight interaction.

**Table 2:** *Enterococcus faecalis* antibacterial activity (in absorbance units, UAbs) of ovalbumin hydrolysates for *Cynara scolymus* proteases. ^1^

MW ^2^	Parameters	Control	Hydrolysis Time (h)		
			2	4	16
TH	Lag phase (min)	719.78 ± 15.7	586.74 ± 8.2 **	598.00 ± 12.6 **	648.94 ± 12.7
	Growth rate (mU Abs/min)	3.73 ± 0.2	2.96 ± 0.2 *	3.15 ± 0.2	3.79 ± 0.2
	Maximum growth (U Abs)	0.566 ± 0.03	0.579 ± 0.01	0.581 ± 0.01	0.551 ± 0.01
<3 kDa	Lag phase (min)	719.78 ± 15.7	554.50 ± 10.24 **	574.88 ± 14.0 **	614.04 ± 21.2 **
	Growth rate (mU Abs/min)	3.73 ± 0.2	2.50 ± 0.1 **	2.65 ± 0.2 **	2.88 ± 0.2 **
	Maximum growth (U Abs)	0.566 ± 0.03	0.555 ± 0.00	0.557 ± 0.01	0.535 ± 0.01

^1^ Values represent means ± SEs (*n* = 3). ^2^ MW = molecular weight, TH = total hydrolysate; <3 kDa = hydrolysate fraction of molecular weight < 3 kDa. *, ** Samples and control were significantly different (Dunnett test, * = *p* < 0.05; ** = *p* < 0.01).

**Table 3:** Peptide sequences identified in ovalbumin hydrolysates from *Cynara scolymus* proteases that were registered in the bioactive peptide database BIOPEP.

Sequence (Origin Hydrolysate)	ID	Origin	MM	Activity
HLFGPPGKKDPV (4 h)	8393	Ovotransferrin	1291.50	ACE-inhibitor
IAAEVYEHTEGSTTSY (2 h, 4 h, 16 h)	8235	Ovotransferrin	1757.81	Antioxidative
PIAAEVYEHTEGSTTSY (2 h)	8231	Ovotransferrin	1854.93	Antioxidative
YAEERYPIL (2 h, 4 h, 16 h)	8391	Ovalbumin	1153.28	ACE-inhibitor

ID = BIOPEP sequence identification number; MM = molecular mass (Da).

## Data Availability

Data is contained within this article.

## References

[1] Miranda J.M., Anton X., Redondo-Valbuena C., Roca-Saavedra P., Rodriguez J.A., Lamas A., Franco C.M., Cepeda A. (2015). Egg and egg-derived foods: Effects on human health and use as functional foods. Nutrients.

[2] Martinez-Villaluenga C., Peñas E., Frias J., Frias J., Martinez-Villaluenga C., Peñas E. (2017). Chapter 2-Bioactive peptides in fermented foods: Production and evidence for health effects. Fermented Foods in Health and Disease Prevention.

[3] Zhang Y., Duan X., Zhuang Y. (2012). Purification and characterization of novel antioxidant peptides from enzymatic hydrolysates of tilapia (*Oreochromis niloticus*) skin gelatin. Peptides.

[4] Zambrowicz A., Eckert E., Pokora M., Bobak Ł., Dąbrowska A., Szołtysik M., Trziszka T., Chrzanowska J. (2015). Antioxidant and antidiabetic activities of peptides isolated from a hydrolysate of an egg-yolk protein by-product prepared with a proteinase from Asian pumpkin (*Cucurbita ficifolia*). RSC Adv..

[5] Lee J.H., Paik H.-D. (2019). Anticancer and immunomodulatory activity of egg proteins and peptides: A review. Poult. Sci..

[6] Lin S., Jin Y., Liu M., Yang Y., Zhang M., Guo Y., Jones G., Liu J., Yin Y. (2013). Research on the preparation of antioxidant peptides derived from egg white with assisting of high-intensity pulsed electric field. Food Chem..

[7] Chen G., Zhang X.W., Benkeblia N. (2012). Proteomics in Food Biotechnology. Omics Technologies: Tools for Food Science.

[8] Yuan J., Zheng Y., Wu Y., Chen H., Tong P., Gao J. (2020). Double enzyme hydrolysis for producing antioxidant peptide from egg white: Optimization, evaluation, and potential allergenicity. J. Food Biochem..

[9] Zhang B., Wang H., Wang Y., Yu Y., Liu J., Liu B., Zhang T. (2019). Identification of antioxidant peptides derived from egg-white protein and its protective effects on H_2_O_2_-induced cell damage. Int. J. Food Sci..

[10] Benedé S., Molina E. (2020). Chicken egg proteins and derived peptides with antioxidant properties. Foods.

[11] Llorente B.E., Brutti C.B., Caffini N.O. (2004). Purification and characterization of a milk-clotting aspartic proteinase from globe artichoke (*Cynara scolymus* L.). J. Agric. Food Chem..

[12] Sidrach L., Garciacanovas F., Tudela J., Neptunorodriguezlopez J. (2005). Purification of cynarases from artichoke (*Cynara scolymus* L.): Enzymatic properties of cynarase A. Phytochemistry.

[13] Tejada L., Abellán A., Cayuela J.M., Martínez-Cacha A., Fernández-Salguero J. (2008). Proteolysis in goats’ milk cheese made with calf rennet and plant coagulant. Int. Dairy J..

[14] Agboola S., Chen S., Zhao J. (2004). Formation of bitter peptides during ripening of ovine milk cheese made with different coagulants. Int. J. Dairy Technol..

[15] Bueno-Gavilá E. (2017). Utilización de Proteasas de Cynara Scolymus L. para la Obtención de Péptidos Bioactivos a partir de Ovoalbúmina, Caseína y leche.

[16] Ren J., Zhao M., Shi J., Wang J., Jiang Y., Cui C., Kakuda Y., Xue S.J. (2008). Purification and identification of antioxidant peptides from grass carp muscle hydrolysates by consecutive chromatography and electrospray ionization-mass spectrometry. Food Chem..

[17] Udenigwe C.C., Aluko R.E. (2012). Food protein-derived bioactive peptides: Production, processing, and potential health benefits. J. Food Sci..

[18] Cheung H.S., Wang F.L., Ondetti M.A., Sabo E.F., Cushman D.W. (1980). Binding of peptide substrates and inhibitors of angiotensin-converting enzyme. Importance of the COOH-terminal dipeptide sequence. J. Biol. Chem..

[19] Nguyen L.T., Haney E.F., Vogel H.J. (2011). The expanding scope of antimicrobial peptide structures and their modes of action. Trends Biotechnol..

[20] Minkiewicz P., Dziuba J., Iwaniak A., Dziuba M., Darewicz M. (2008). BIOPEP database and other programs for processing bioactive peptide sequences. J. AOAC Int..

[21] Fernández-Salguero J., Tejada L., Gómez R. (2002). Use of powdered vegetable coagulant in the manufacture of ewe’s milk cheeses: Cheese-making with a powdered vegetable coagulant. J. Sci. Food Agric..

[22] Association of Analytical Chemists (1990). Official Methods of Analysis.

[23] González de Llano D., Polo M.C., Ramos M. (1994). Study of proteolysis in artisanal cheeses: High performance liquid chromatography of peptides. J. Dairy Sci..

[24] Cushman D.W., Cheung H.S. (1971). Spectrophotometric assay and properties of the angiotensin-converting enzyme of rabbit lung. Biochem. Pharmacol..

[25] Miguel M., Recio I., Gómez-Ruiz J.A., Ramos M., López-Fandiño R. (2004). Angiotensin I-converting enzyme inhibitory activity of peptides derived from egg white proteins by enzymatic hydrolysis. J. Food Prot..

[26] Bersuder P., Hole M., Smith G. (1998). Antioxidants from heated histidine-glucose model system. I: Investigation of the antioxidant role of histidine and isolation of antioxidants by high-performance liquid chromatography. J. Am. Oil Chem. Soc..

[27] De Gobba C., Espejo-Carpio F.J., Skibsted L.H., Otte J. (2014). Antioxidant peptides from goat milk protein fractions hydrolysed by two commercial proteases. Int. Dairy J..

[28] Wu C.-R., Huang M.-Y., Lin Y.-T., Ju H.-Y., Ching H. (2007). Antioxidant properties of Cortex Fraxini and its simple coumarins. Food Chem..

[29] Hill L.E., Gomes C., Taylor T.M. (2013). Characterization of beta-cyclodextrin inclusion complexes containing essential oils (trans-cinnamaldehyde, eugenol, cinnamon bark, and clove bud extracts) for antimicrobial delivery applications. LWT-Food Sci. Technol..

[30] Bueno-Gavilá E., Abellán A., Girón-Rodríguez F., Cayuela J.M., Salazar E., Gómez R., Tejada L. (2019). Bioactivity of hydrolysates obtained from bovine casein using artichoke (*Cynara scolymus* L.) proteases. J. Dairy Sci..

[31] Shen S., Chahal B., Majumder K., You S.-J., Wu J. (2010). Identification of novel antioxidative peptides derived from a thermolytic hydrolysate of ovotransferrin by LC-MS/MS. J. Agric. Food Chem..

[32] Konrad B., Anna D., Marek S., Marta P., Aleksandra Z., Józefa C. (2014). The evaluation of dipeptidyl peptidase (DPP)-IV, α-glucosidase and angiotensin converting enzyme (ACE) inhibitory activities of whey proteins hydrolyzed with serine protease isolated from asian pumpkin (*Cucurbita ficifolia*). Int. J. Pept. Res. Ther..

[33] Duan X., Wu F., Li M., Yang N., Wu C., Jin Y., Yang J., Jin Z., Xu X. (2014). Naturally occurring angiotensin I-converting enzyme inhibitory peptide from a fertilized egg and its inhibitory mechanism. J. Agric. Food Chem..

[34] Garcés-Rimón M., López-Expósito I., López-Fandiño R., Miguel M. (2016). Egg white hydrolysates with in vitro biological multiactivities to control complications associated with the metabolic syndrome. Eur. Food Res. Technol..

[35] Chen C., Chi Y.-J. (2011). Antioxidant, ACE inhibitory activities and functional properties of egg white protein hydrolysate. J. Food Biochem..

[36] Huang Q., Li S., Teng H., Jin Y., Ma M., Song H. (2015). Optimizing preparation conditions for angiotensin-I-converting enzyme inhibitory peptides derived from enzymatic hydrolysates of ovalbumin. Food Sci. Biotechnol..

[37] Jakovetić S., Luković N., Jugović B., Gvozdenović M., Grbavčić S., Jovanović J., Knežević-Jugović Z. (2015). Production of antioxidant egg white hydrolysates in a continuous stirred tank enzyme reactor coupled with membrane separation unit. Food Bioprocess Technol..

[38] Chiang W.-D., Tsou M.-J., Weng C.-H., Tsai T.-C. (2008). Production of angiotensin I-converting enzyme inhibitor derived from egg white protein hydrolysates using a membrane reactor. J. Food Drug Anal..

[39] Chen C., Chi Y.-J., Zhao M.-Y., Xu W. (2012). Influence of degree of hydrolysis on functional properties, antioxidant and ACE inhibitory activities of egg white protein hydrolysate. Food Sci. Biotechnol..

[40] Sun S., Niu H., Yang T., Lin Q., Luo F., Ma M. (2014). Antioxidant and anti-fatigue activities of egg white peptides prepared by pepsin digestion: Antioxidant and anti-fatigue activities of egg white peptides. J. Sci. Food Agric..

[41] You S.-J., Wu J. (2011). Angiotensin-I Converting Enzyme Inhibitory and Antioxidant Activities of Egg Protein Hydrolysates Produced with Gastrointestinal and Nongastrointestinal Enzymes. J. Food Sci..

[42] Liu J., Jin Y., Lin S., Jones G.S., Chen F. (2015). Purification and identification of novel antioxidant peptides from egg white protein and their antioxidant activities. Food Chem..

[43] Kobayashi Y., Yamauchi T., Katsuda T., Yamaji H., Katoh S. (2008). Angiotensin-I converting enzyme (ACE) inhibitory mechanism of tripeptides containing aromatic residues. J. Biosci. Bioeng..

[44] Guo H., Kouzuma Y., Yonekura M. (2009). Structures and properties of antioxidative peptides derived from royal jelly protein. Food Chem..

[45] Quirós A., Chichón R., Recio I., López-Fandiño R. (2007). The use of high hydrostatic pressure to promote the proteolysis and release of bioactive peptides from ovalbumin. Food Chem..

[46] Tanzadehpanah H., Asoodeh A., Saberi M.R., Chamani J. (2013). Identification of a novel angiotensin-I converting enzyme inhibitory peptide from ostrich egg white and studying its interactions with the enzyme. Innov. Food Sci. Emerg. Technol..

[47] Pokora M., Zambrowicz A., Dąbrowska A., Eckert E., Setner B., Szołtysik M., Szewczuk Z., Zabłocka A., Polanowski A., Trziszka T. (2014). An attractive way of egg white protein by-product use for producing of novel anti-hypertensive peptides. Food Chem..

[48] Abeyrathne E.D.N.S., Lee H.Y., Jo C., Nam K.C., Ahn D.U. (2014). Enzymatic hydrolysis of ovalbumin and the functional properties of the hydrolysates. Poult. Sci..

[49] Zambrowicz A., Timmer M., Eckert E., Trziszka T. (2013). Evaluation of the ACE-Inhibitory Activity of Egg-White Proteins Degraded with Pepsin. Pol. J. Food Nutr. Sci..

[50] Abubakar A., Saito T., Kitazawa H., Kawai Y., Itoh T. (1998). Structural analysis of new antihypertensive peptides derived from cheese whey protein by proteinase K digestion. J. Dairy Sci..

[51] Mullally M.M., Meisel H., FitzGerald R.J. (1997). Angiotensin-I-converting enzyme inhibitory activities of gastric and pancreatic proteinase digests of whey proteins. Int. Dairy J..

[52] Mullally M.M., Meisel H., FitzGerald R.J. (1997). Identification of a novel angiotensin-I-converting enzyme inhibitory peptide corresponding to a tryptic fragment of bovine β-lactoglobulin. FEBS Lett..

[53] Galán E., Prados F., Pino A., Tejada L., Fernández-Salguero J. (2008). Influence of different amounts of vegetable coagulant from cardoon *Cynara cardunculus* and calf rennet on the proteolysis and sensory characteristics of cheeses made with sheep milk. Int. Dairy J..

[54] Natesh R., Schwager S.L.U., Sturrock E.D., Acharya K.R. (2003). Crystal structure of the human angiotensin-converting enzyme–lisinopril complex. Nature.

[55] Lee D., Bamdad F., Khey K., Sunwoo H.H. (2017). Antioxidant and anti-inflammatory properties of chicken egg vitelline membrane hydrolysates. Poult. Sci..

[56] Lozano-Ojalvo D., Molina E., López-Fandiño R. (2016). Regulation of exacerbated immune responses in human peripheral blood cells by hydrolysed egg white proteins. PLoS ONE.

[57] Chen C., Chi Y.-J., Zhao M.-Y., Lv L. (2012). Purification and identification of antioxidant peptides from egg white protein hydrolysate. Amino Acids.

[58] Chen H., Muramoto K., Yamauchi F., Fujimoto K., Nokihara K. (1998). Antioxidative properties of histidine-containing peptides designed from peptides fragments found in the digests of a soybean protein. J. Agric. Food Chem..

[59] Homayouni-Tabrizi M., Asoodeh A., Abbaszadegan M.-R., Shahrokhabadi K., Nakhaie Moghaddam M. (2015). An identified antioxidant peptide obtained from ostrich (*Struthio camelus*) egg white protein hydrolysate shows wound healing properties. Pharm. Biol..

[60] Noh D.O., Suh H.J. (2015). Preparation of egg white liquid hydrolysate (ELH) and its radical-scavenging activity. Prev. Nutr. Food Sci..

[61] Baratzadeh M.-H., Asoodeh A., Chamani J. (2013). Antioxidant peptides obtained from goose egg white proteins by enzymatic hydrolysis. Int. J. Food Sci..

[62] Zhu L., Chen J., Tang X., Xiong Y.L. (2008). Reducing, Radical Scavenging, and Chelation Properties of in Vitro Digests of Alcalase-Treated Zein Hydrolysate. J. Agric. Food Chem..

[63] Chen H., Yamauchi F., Nokihara K. (1996). Antioxidant activity of designed peptides based on the antioxidative peptide isolated from digests of a soybean protein. J. Agric. Food Chem..

[64] Yu Z., Yin Y., Zhao W., Chen F., Liu J. (2014). Application and bioactive properties of proteins and peptides derived from hen eggs: Opportunities and challenges: Application of proteins and peptides from hen eggs. J. Sci. Food Agric..

[65] Memarpoor-Yazdi M., Asoodeh A., Chamani J. (2012). A novel antioxidant and antimicrobial peptide from hen egg white lysozyme hydrolysates. J. Funct. Foods.

[66] Thammasirirak S., Pukcothanung Y., Preecharram S., Daduang S., Patramanon R., Fukamizo T., Araki T. (2010). Antimicrobial peptides derived from goose egg white lysozyme. Comp. Biochem. Physiol. C Toxicol. Pharmacol..

[67] Mine Y., Ma F., Lauriau S. (2004). Antimicrobial peptides released by enzymatic hydrolysis of hen egg white lysozyme. J. Agric. Food Chem..

[68] Tang W., Zhang H., Wang L., Qian H. (2013). Antimicrobial peptide isolated from ovalbumin hydrolysate by immobilized liposome-binding extraction. Eur. Food Res. Technol..

[69] Asoodeh A., Homayouni-Tabrizi M., Shabestarian H., Emtenani S., Emtenani S. (2016). Biochemical characterization of a novel antioxidant and angiotensin I-converting enzyme inhibitory peptide from Struthio camelus egg white protein hydrolysis. J. Food Drug Anal..

